# Ginkgolide B Suppresses TLR4-Mediated Inflammatory Response by Inhibiting the Phosphorylation of JAK2/STAT3 and p38 MAPK in High Glucose-Treated HUVECs

**DOI:** 10.1155/2017/9371602

**Published:** 2017-07-12

**Authors:** Kun Chen, Wenjia Sun, Yun Jiang, Beidong Chen, Yanyang Zhao, Jie Sun, Huan Gong, Ruomei Qi

**Affiliations:** The MOH Key Laboratory of Geriatrics, Beijing Hospital, National Center of Gerontology, Beijing, China

## Abstract

**Aim:**

Ginkgolide B is a *Ginkgo biloba* leaf extract that has been identified as a natural platelet-activating factor receptor (PAFR) antagonist. We investigated the effect of ginkgolide B on high glucose-induced TLR4 activation in human umbilical vein endothelial cells (HUVECs).

**Methods:**

Protein expression was analyzed by immunoblotting. Small-interfering RNA (siRNA) was used to knock down PAFR and TLR4 expression.

**Results:**

Ginkgolide B suppressed the expression of TLR4 and MyD88 that was induced by high glucose. Ginkgolide B also reduced the levels of platelet endothelial cell adhesion molecule-1, interleukin-6, and monocyte chemotactic protein 1. Further, we examined the association between PAFR and TLR4 by coimmunoprecipitation. The result showed that high glucose treatment caused the binding of PAFR and TLR4, whereas ginkgolide B abolished this binding. The functional analysis indicated that PAFR siRNA treatment reduced TLR4 expression, and TLR4 siRNA treatment decreased PAFR expression in high glucose-treated HUVECs, further supporting the coimmunoprecipitation data. Ginkgolide B inhibited the phosphorylation of Janus kinase 2 (JAK2)/signal transducer and activator of transcription 3 (STAT3) and p38 mitogen-activated protein kinase (MAPK).

**Conclusion:**

Ginkgolide B exerted protective effects by inhibiting the TLR4-mediated inflammatory response in high glucose-treated endothelial cells. The mechanism of action of ginkgolide B might be associated with inhibition of the JAK2/STAT3 and p38 MAPK phosphorylation.

## 1. Introduction

Diabetes is the most important risk factor for cardiovascular disease, which is the leading cause of morbidity and mortality in patients with diabetes. Macrovascular and microvascular damage is the main pathological characteristic of diabetic complications. However, the molecular mechanism of vascular inflammatory injury in diabetes remains unclear. Growing evidence demonstrates that toll-like receptors (TLRs) of the innate immune system are involved in the pathological process of diabetes [1–3]. Toll-like receptor activation can be triggered by external pathogens and endogenous harmful products, such as oxidative stress, the excessive accumulation of lipids, and tissue- or cell-derived inflammatory factors (i.e., damage-associated molecular patterns) [4–6]. The TLR family has 10 subtypes in humans and 12 subtypes in mice [7]. Toll-like receptors are abundantly expressed in polymorphonuclear cells, macrophages, T cells, and B cells. Moreover, TLR expression has been found in cardiac, endothelial, and vascular smooth muscle cells [8]. Several lines of evidence have revealed that TLR activation, particularly TLR4, is linked to atherosclerosis in diabetes [9, 10]. Several recent studies reported that TLR4 levels increased in patients with type 2 diabetes, suggesting that a high glucose concentration results in TLR4 activation in diabetes [11–13]. However, the precise mechanism of action of TLR signaling and its potential functions remains largely unknown.

Platelet-activating factor (PAF) is a potent phospholipid inflammatory mediator. By binding PAF receptor (PAFR), PAF elicits inflammatory responses in various cell types. A recent study reported that the activity of PAF-acetylhydrolase (PAFAH; an enzyme that catabolizes PAF) increased in hypercholesterolemic minipigs [14]. Platelet-activating factor enhanced matrix metalloproteinase-2 production in vascular smooth muscle cells through a *β*-arrestin-dependent extracellular signal-regulated kinase (ERK) signaling pathway [15]. Agrawal et al. recently reported that PAF signaling was associated with TLR4 in peritoneal macrophages in mice [16]. Moreover, a PAFR antagonist exerted cardioprotective effects in myocardial ischemia/reperfusion [17]. Our previous studies showed that the PAFR inhibitor ginkgolide B reduced the plaque area and vascular inflammation in apolipoprotein E-deficient mice [18]. These results suggest that inhibiting PAFR-mediated signaling might be a potential strategy for preventing diabetic complications.

Signal transducer and activator of transcription (STAT) proteins are a family of latent transcription factors that are activated by cytokines and growth factors. Mammals possess seven STAT proteins; of these, STAT3 is a multifunctional member that is involved in the acute-phase response, development, cell growth and differentiation, and immunity. STAT3 is phosphorylated by Janus family kinase (JAK) at Tyr^705^ and dimerizes and translocates to the nucleus to regulate gene expression [19]. The activation of receptor and nonreceptor tyrosine kinases stimulates STAT3 Tyr^705^ to induce dimerization and increase STAT3 DNA-binding activity [20]. The phosphorylation of Ser^727^ is mediated by various serine kinases, such as MAPKs, cyclin-dependent kinases, and protein kinase C, and this modification increases the transcriptional activity of STAT3 by facilitating protein-protein interactions with transcriptional coactivators [21]. A recent study showed that TLR4 signaling promoted a cyclooxygenase-1/prostaglandin E2/STAT3-positive feedback loop in hepatocellular carcinoma cells [22]. However, unknown is whether STAT3 is involved in high glucose-induced TLR4 activation in endothelial cells.

Ginkgolide B is a *Ginkgo biloba* leaf extract that has been identified as a natural PAFR antagonist [23]. Our previous studies revealed that ginkgolide B decreased inflammatory protein expression in oxidized low-density lipoprotein-stimulated endothelial cells and inhibited platelet release inflammatory mediators [24, 25]. However, remaining unknown is whether ginkgolide B can inhibit the high glucose-induced TLR4-mediated inflammatory response. The present study investigated the effect of ginkgolide B on TLR4-mediated signaling and the underlying mechanisms in high glucose-treated human umbilical vein endothelial cells (HUVECs).

## 2. Materials and Methods

### 2.1. Ethics Statement

According to the Declaration of Helsinki, the umbilical cords were donated by cesarean section patients, from whom we received written informed consent. The study was approved by the Ethics Committee of the Beijing Institute of Geriatrics (number 201420).

### 2.2. Materials

Ginkgolide B (95% purity) was purchased from Daguanyuan Company (Xuzhou, Jiangsu, China). Monoclonal anti-TLR4 antibody was purchased from Abcam (Boston, MA, USA). Polyclonal anti-PAFR antibody was purchased from Cayman Chemical (Ann Arbor, MI, USA). Monoclonal polyclonal anti-platelet endothelial cell adhesion molecule-1 (PECAM-1) antibodies, polyclonal anti-phosphorylated STAT3 antibodies, anti-STAT3 antibodies, and monoclonal anti-*β*-actin antibodies were purchased from Santa Cruz Biotechnology (Santa Cruz, CA, USA). Polyclonal anti-p38 MAPK, polyclonal anti-phosphorylated p38 MAPK, monoclonal anti-ERK, and monoclonal anti-phosphorylated ERK antibodies were purchased from Cell Signaling Technologies (Danvers, MA, USA). Dylight 594 goat anti-mouse antibodies were purchased from Zhongshan Jinqiao Biotechnology (Zhongshan Jinqiao, Beijing, China).

### 2.3. Preparation and Culture of HUVECs

Primary HUVECs were collected from human umbilical veins by digestion in 0.1% collagenase I in M199 medium (Gibco, NY, USA) for 15 min. The solution was collected and centrifuged at 1000 rotations per minute (rpm) for 10 min. The cells were cultured in M199 medium that contained 10% fetal bovine serum (Gibco, NY, USA), 2 mM glutamine, 100 U/ml penicillin, 100 *μ*g/ml streptomycin, and 20 ng/ml endothelial growth factor (R&D Minneapolis, MN, USA) in an incubator at 37°C and 5% CO_2_. HUVECs at passage 3 were used in the present study.

### 2.4. Immunoblotting and Immunoprecipitation

HUVECs were incubated with various concentrations of ginkgolide B (0.2, 0.4, and 0.6 mg/ml) for 1 h and then exposed to 30 mM glucose for 8 h. Cell lysis was performed in lysis buffer (1% Triton X-100, 100 mM Tris/HCl [pH 7.2], 50 mM NaCl, 5 mM ethylenediaminetetraacetic acid (EDTA), 5 mM ethylene glycol tetraacetic acid (EGTA), 1 *μ*M phenylmethylsulfonyl fluoride (PMSF), and 100 *μ*g/ml leupeptin) and then centrifuged at 15000 ×g at 4°C for 5 min. Protein samples were boiled in sodium dodecyl sulfate (SDS) loading buffer for 5 min, run on SDS-polyacrylamide gel electrophoresis (PAGE), and transferred to a polyvinylidene difluoride (PVDF) membrane. Primary antibody incubations were performed overnight at 4°C. Horseradish peroxidase-conjugated secondary antibody was applied for 1 h at room temperature and developed using Super Signal developing reagent (Pierce, Thermo Fisher Scientific). Blot densitometry was then performed, and the bands were analyzed using a Gene Genius Bio Imaging System.

For immunoprecipitation, the cells were incubated with or without ginkgolide B for 1 h, and then high glucose (30 mM) was added for 8 h. HUVEC lysates were incubated with antibodies against PAFR or TLR4 overnight at 4°C, followed by incubation with protein A/G Sepharose beads for 2 h at 4°C. The samples were centrifuged at 800 ×g for 5 min to collect protein A/G beads, which were washed four times and boiled in SDS buffer. Eluates from the beads were separated by 8% SDS-PAGE and immunoblotted for PAFR, TLR4, and *β*-actin. Nonimmune isotype immunoglobulin G was used for immunoprecipitation as a negative control.

### 2.5. Reverse Transcription Polymerase Chain Reaction

TLR4 mRNA expression was detected by reverse transcription polymerase chain reaction (RT-PCR). Cells were treated with various doses of ginkgolide B for 1 h, and high glucose was added for another 8 h. Total RNAs were isolated for HUVEC culturing using Trizol reagent (Thermo Fisher Scientific, Waltham, USA). RT-PCR was performed using SYBR Premix Ex Taq mix (Takara, Dalian, China). The primers were the following: forward (5′-CCGCTTCCTGGTCTTATCAT) and reverse (5′-TCTGCTGCAACTCATTTCAT). After amplification, portions of the PCR mixtures were electrophoresed on a 2% agarose gel and visualized by a Bio-Rad Gel Doc 2000 device (Bio-Rad, Hercules, CA, USA).

### 2.6. Interleukin-6 and Monocyte Chemotactic Protein 1 Enzyme-Linked Immunosorbent Assay

For the detection of interleukin-6 (IL-6) and monocyte chemotactic protein 1 (MCP-1) in the supernatant, cells were plated in 24-well plates. The cells were then treated with and without ginkgolide B for 1 h, and then high glucose (30 mM) was added for 8 h. After centrifugation at 1000 rpm for 10 min, the supernatant was collected to measure IL-6 and MCP-1. The levels of secreted IL-6 and MCP-1 were determined by an enzyme-linked immunosorbent assay (ELISA) kit (NeoBioscience, Xinbosheng, China) according to the manufacturer's instructions.

### 2.7. Immunofluorescent Staining of TLR4 and STAT3

HUVECs were cultured on six-well culture plates. The cells were pretreated with ginkgolide B for 1 h and then with high glucose (30 mM) for 8 h. The cells were fixed with 4% paraformaldehyde for 15 min and washed twice. The cells were then incubated with antibody against TLR4 or STAT3 for 1.5 h. After washing three times, Dylight-conjugated antibody was added to the cells for 1.5 h at room temperature. The nucleus was stained by DAPI for 5 min at room temperature, and staining was observed under a fluorescence microscope.

### 2.8. Small-Interfering RNA against PAFR and TLR4

HUVECs were cotransfected with Lipofectamine RNAiMAX Transfection Reagent (Life Technologies) that contained PAFR-selective small-interfering RNA (siRNA) or TLR4 siRNA for 48 h. The siRNA PAFR sequence was 5′-GGCCAUUAAUGAUGCACAU(dTdT)-3′ [26]. PAFR siRNA was synthesized by RiboBio (Guangzhou, China). TLR4 siRNA was purchased from Santa Cruz Biotechnology (Catalog number sc-40260, Santa Cruz, CA, USA). Immunoblotting was performed to examine the efficiency of protein knockdown for PAFR and TLR4.

### 2.9. Statistical Analysis

Quantitative data are presented as mean ± SEM. Significant differences between two groups were analyzed by two-tail unpaired Student's *t*-test. All of the calculations were performed using SPSS 18.0 software (Armonk, NY, USA). Values of *p* < 0.05 were considered statistically significant.

## 3. Results

### 3.1. Ginkgolide B Inhibited the Expression of TLR and MyD88 that Was Induced by High Glucose

**Figure 1:**
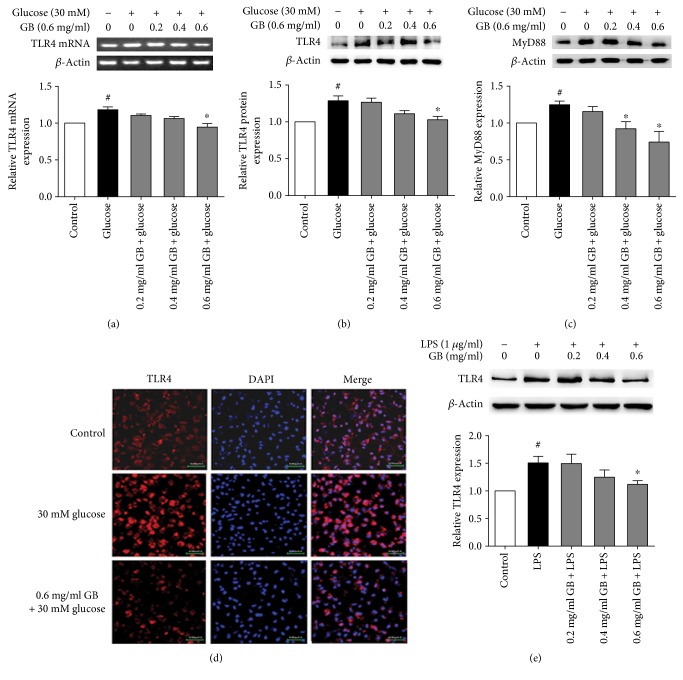
Ginkgolide B inhibits TLR4 expression in high glucose- and LPS-treated HUVECs. The cells were treated with various concentrations of ginkgolide B (GB) for 1 h, and glucose (30 mM) or LPS (1 *μ*g/ml) was then added for 8 h. (a) Ginkgolide B suppressed TLR4 RNA expression that was induced by high glucose. (b) Ginkgolide B decreased TLR4 protein expression that was induced by high glucose. (c) Ginkgolide B decreased MyD88 expression that was induced by high glucose. (d) TLR4 expression was detected by immunofluorescence in high glucose-treated HUVECs. (e) Ginkgolide B suppressed TLR4 expression that was induced by LPS. The data were obtained from four independent experiments. The column chart represents the density analysis from four independent experiments. ^#^*p* < 0.05, significant difference between nonhigh glucose-treated cells and high glucose cells (a–c) or between non-LPS-treated cells and LPS-treated cells (e); ^∗^*p* < 0.05, significant difference between non-ginkgolide B-treated cells and ginkgolide B-treated cells.

TLR4 plays an important role in diabetes and diabetic complications. To determine the effects of ginkgolide B on the TLR4-mediated inflammatory response in high glucose-treated HUVECs, we investigated the effects of ginkgolide B on the expression of TLR4 and MyD88, which is a downstream molecule of the TLR4 signaling pathway. The cells were incubated with various doses of ginkgolide B (0.2, 0.4, and 0.6 mg/ml) for 1 h and then exposed to 30 mM glucose for 8 h. Semiquantitative RT-PCR was performed to determine TLR4 mRNA expression. As shown in Figures 1(a) and 1(b), 30 mM glucose treatment increased TLR4 mRNA levels by 18.1%. Ginkgolide B dose dependently decreased TLR4 mRNA expression. TLR4 protein expression was then examined. High glucose treatment increased TLR4 protein expression by 28.7%, and ginkgolide B abolished the high glucose-induced increase in TLR4 expression. To determine whether the high glucose-induced inflammatory response is mediated in TLR4 signaling, we evaluated MyD88 expression by Western blot, which is a downstream molecule of TLR4. As shown in Figure 1(c), high glucose treatment increased MyD88 expression by 24.8%, and ginkgolide B dose dependently attenuated this expression. An immunofluorescence experiment was performed to detect TLR4 expression in high glucose-treated HUVECs. As shown in Figure 1(d), the immunofluorescence intensity of TLR was enhanced by high glucose treatment, and 0.6 mg/ml ginkgolide B attenuated this effect. To further determine the effect of ginkgolide B inhibition of TLR4 activation, we used the TLR4 ligand lipopolysaccharide (LPS). As shown in Figure 1(e), ginkgolide B (0.6 mg/ml) significantly abolished TLR4 expression that was induced by LPS.

### 3.2. Ginkgolide B Reduced the Levels of PECAM-1, IL-6, and MCP-1 in High Glucose-Treated HUVECs

**Figure 2:**
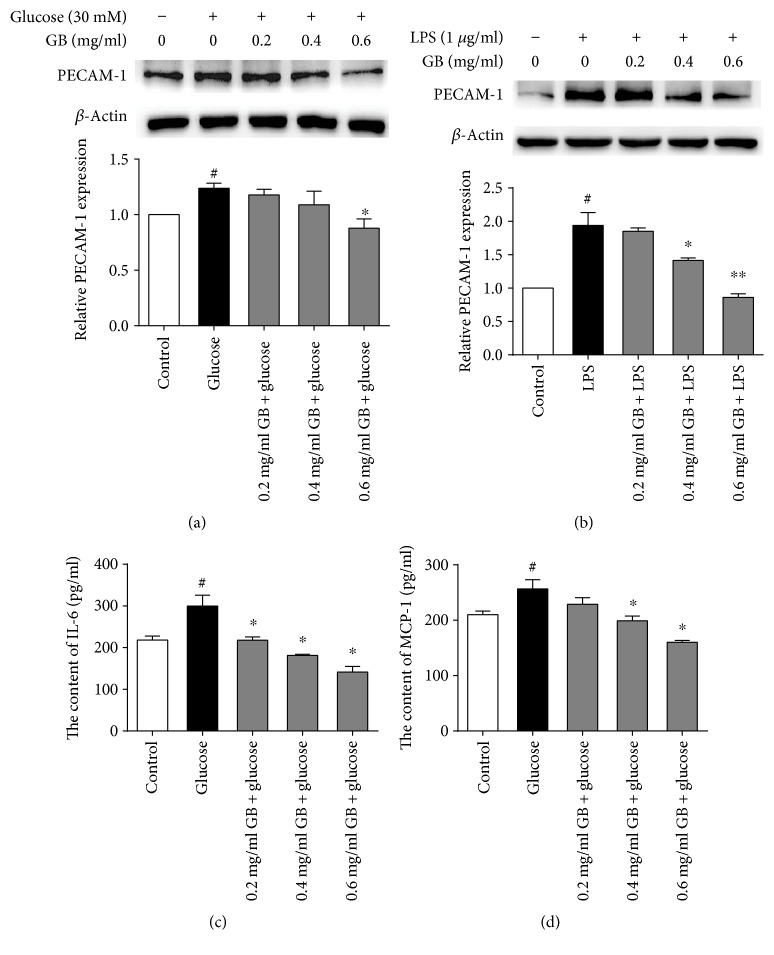
Ginkgolide B decreases PECAM-1 expression and the levels of IL-6 and MCP-1 in high glucose-treated HUVECs. The cells were treated with various concentrations of ginkgolide B for 1 h, and glucose or LPS was added for another 8 h. Protein expression was analyzed by Western blot. The column chart represents the density analysis from four independent experiments. (a) Ginkgolide B reduced PECAM-1 expression that was induced by high glucose. (b) Ginkgolide B decreased PECAM-1 expression that was induced by LPS. (c) Ginkgolide B reduced the levels of IL-6 in high glucose-treated cells. (d) Ginkgolide B reduced the levels of MCP-1 in high glucose-treated cells. ^#^*p* < 0.05, significant difference between nonhigh glucose-treated cells and high glucose-treated cells (a) or between non-LPS-treated cells and LPS-treated cells (b); ^∗^*p* < 0.05, significant difference compared non-ginkgolide B-treated cells and ginkgolide B-treated cells; ^∗∗^*p* < 0.01, significant difference compared non-ginkgolide B-treated cells and ginkgolide B-treated cells.

To evaluate the effects of ginkgolide B on inflammatory protein expression in high glucose-treated HUVECs, PECAM-1 expression was detected by Western blot. As shown in Figure 2(a), high glucose treatment increased PECAM-1 expression by 23.7%, and ginkgolide B attenuated this increase in PECAM-1 expression. To confirm that the inhibitory effect of ginkgolide B on PECAM-1 expression was mediated by TLR4 activation in high glucose-treated HUVECs, we detected PECAM-1 expression that was induced by LPS. As shown in Figure 2(b), ginkgolide B (0.4 and 0.6 mg/ml) suppressed PECAM-1 expression in LPS-treated cells. Next, we evaluated the effect of ginkgolide B on secretion of the inflammatory proteins IL-6 and MCP-1 by ELISA. As shown in Figures 2(c) and 2(d), the levels of IL-6 were 299.75 ± 45.43 pg/ml in the high glucose-treated group and 141.56 ± 22.81 pg/ml in the ginkgolide B-treated group, with a significant difference between groups. The levels of MCP-1 were also increased by high glucose treatment. The levels of MCP-1 were 256.5 ± 28.91 pg/ml in high glucose-treated cells and 160.1 ± 6.22 pg/ml in ginkgolide B-treated cells, with a significant difference between groups.

### 3.3. High Glucose Increased the Binding between PAFR and TLR4, and Ginkgolide B Decreased Their Expression in HUVECs

**Figure 3:**
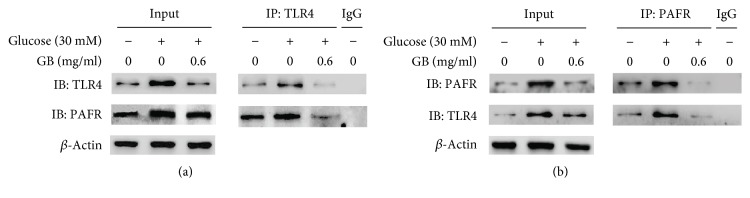
Ginkgolide B inhibits high glucose-induced increase in the association between PAFR and TLR4. HUVECs were treated with 0.6 mg/ml ginkgolide B for 1 h, and glucose was then added for another 8 h. Coimmunoprecipitation (Co-IP) was performed using an anti-TLR4 antibody or an anti-PAFR antibody. Rabbit IgG served as a nonimmune control. (a) PAFR expression was determined by immunoprecipitation with anti-TLR4 antibody. (b) TLR4 expression was determined by immunoprecipitation with anti-PAFR antibody. The input represents whole cell lysate. The data were obtained from three independent experiments.

Our previous studies found that high glucose treatment increased PAFR expression in HUVECs. We hypothesized that PAFR might be associated with TLR4 under condition of high glucose stimulation. We performed coimmunoprecipitation (Co-IP) experiments using anti-TLR4 antibody and anti-PAFR antibody. The results showed that anti-TLR4 antibody immunoprecipitated TLR4 and PAFR (Figure 3(a)), and anti-PAFR antibody immunoprecipitated PAFR and TLR4 in high glucose-stimulated HUVECs (Figure 3(b)). Ginkgolide B decreased the binding of PAFR and TLR4 in high glucose-treated cells.

### 3.4. Effect of siRNA against PAFR on TLR4 Expression in HUVECs

**Figure 4:**
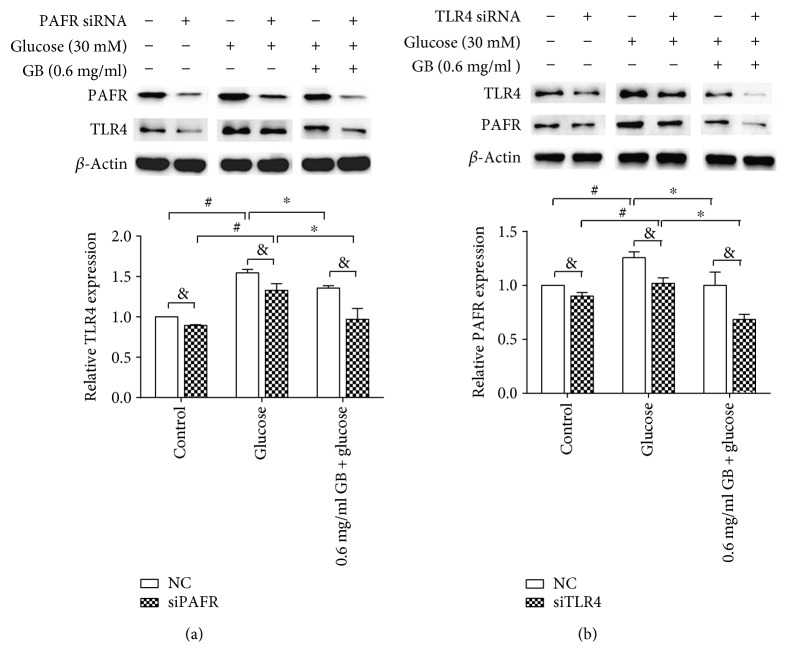
Synergistic inhibitory effect of ginkgolide B on the expression of TLR4 and PAFR in PAFR siRNA- and TLR4 siRNA-treated HUVECs. HUVECs were transfected by siRNA against PAFR or siRNA against TLR4 for 48 h to knock down PAFR or TLR4. The HUVECs were treated with 0.6 mg/ml ginkgolide B for 1 h, and glucose was then added for another 8 h. TLR4 and PFAR expression was analyzed by Western blot. (a) TLR4 expression in PAFR siRNA-transfected cells. (b) PAFR expression in TLR4 siRNA-transfected cells. Data for the density analysis were obtained from three independent experiments. ^&^*p* < 0.05, significant difference between negative siRNA-transfected cells (negative control (NC)) and PAFR siRNA-transfected cells or TLR4 siRNA-transfected cells; ^#^*p* < 0.05, significant difference between nonhigh glucose-treated cells and high glucose-treated cells; ^∗^*p* < 0.05, significant difference between non-ginkgolide B-treated cells and ginkgolide B-treated cells.

To further determine the interaction between PAFR and TLR4 in high glucose-treated HUVECs, we conducted a functional analysis using siRNA to knock down the PAFR and TLR4 genes. HUVECs were transfected with siRNA against PAFR and a negative control siRNA for 48 h, and a high glucose concentration (30 mM) was added to the cells for another 8 h. As shown in Figure 4(a), treatment with PAFR siRNA reduced TLR4 expression by 9.9% and 23.8% in nonhigh glucose-treated cells and high glucose-treated cells, respectively, compared with the negative control, with a significant difference between the negative control and PAFR siRNA-transfected cells. Ginkgolide B exerted a more potent inhibitory effect on high glucose-induced TLR4 expression in PAFR siRNA-transfected cells compared with the negative control. We also evaluated the effects of TLR4 siRNA on PAFR expression. As shown in Figure 4(b), treatment with TLR4 siRNA reduced PAFR expression by 9.1% and 19.2% in nonhigh glucose-treated cells and high glucose-treated cells, respectively, compared with the negative control. Ginkgolide B exerted a synergistic inhibitory effect on PAFR expression that was induced by TLR4 siRNA treatment, with a significant difference between the negative control and TLR4 siRNA-transfected cells.

### 3.5. Ginkgolide B Abolished JAK2/STAT3 Phosphorylation Induced by High Glucose

**Figure 5:**
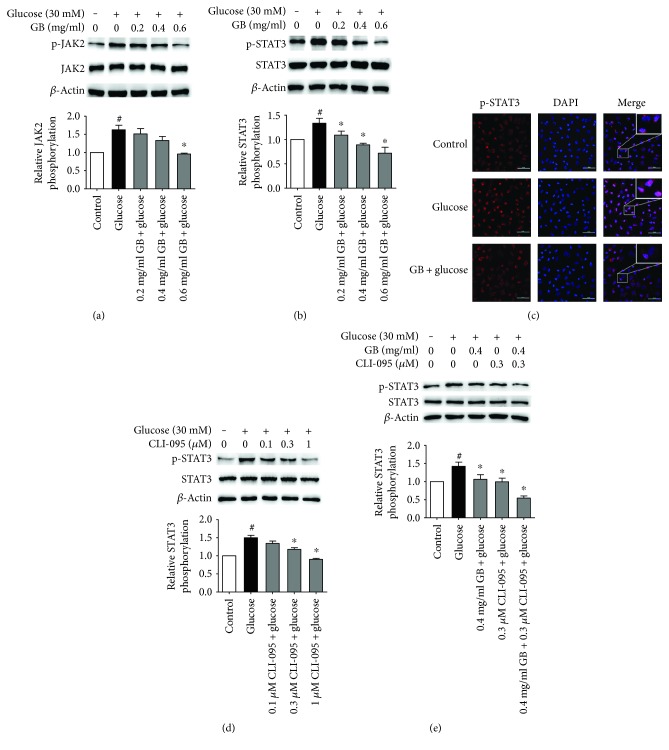
Ginkgolide B inhibits the phosphorylation of JAK2 and STAT3 in high glucose-treated HUVECs. The cells were treated with various concentrations of ginkgolide B for 1 h, and glucose was added for another 8 h. Phosphorylated JAK2 and STAT3 were analyzed by Western blot. (a, b) Ginkgolide B inhibited JAK2 and STAT3 phosphorylation that was induced by high glucose. (c) Phosphorylated STAT3 expression was determined by immunofluorescence. (d) CLI-095 inhibited STAT3 phosphorylation that was induced by LPS. (e) The combination of ginkgolide B and CLI-095 synergistically inhibited STAT3 phosphorylation that was induced by high glucose. ^#^*p* < 0.05, significant difference between nonhigh glucose-treated cells and high glucose-treated cells; ^∗^*p* < 0.05, significant difference between non-ginkgolide B-treated cells and ginkgolide B-treated cells.

TLR4 signaling is linked to nuclear transcription factor-*κ*B activation and the inflammatory response [27, 28]. However, whether STAT3 is involved in TLR4 signaling in high glucose-stimulated endothelial cells remains unknown. To investigate whether the effect of ginkgolide B on the inhibition of TLR4 is related to JAK2/STAT3, we detected the phosphorylation of JAK2 and STAT3 Tyr^705^. As shown in Figures 5(a) and 5(b), JAK2 phosphorylation increased by 62.7% in high glucose-treated HUVECs, and ginkgolide B dose dependently inhibited JAK2 phosphorylation. High glucose (30 mM) stimulation also increased STAT3 phosphorylation by 30.5%, and 0.6 mg/ml ginkgolide B completely abolished STAT3 phosphorylation. Next, phosphorylated STAT3 expression was detected by immunofluorescence. As shown in Figure 5(c), the level of phosphorylated STAT3 was higher in the nucleus in high glucose-treated cells than in controls. The level of phosphorylated STAT3 in the nucleus in ginkgolide B-treated cells was similar to controls. Moreover, to further confirm that TLR4 activation is linked to STAT3, we evaluated the effect of the TLR4 inhibitor CLI-095 on STAT3 phosphorylation. As shown in Figure 5(d), CLI-095 (0.1, 0.3, and 1 *μ*M) attenuated STAT3 phosphorylation that was induced by high glucose, and 1 *μ*M CLI-095 completely inhibited STAT3 phosphorylation. To determine the inhibitory effect of ginkgolide B on STAT3 phosphorylation is associated with TLR4 activation, we then investigated whether combined CLI-095 and ginkgolide B synergistically inhibits STAT3 phosphorylation. As shown in Figure 5(e), separate treatment with 0.4 mg/ml ginkgolide B or 0.3 *μ*M CLI-095 alone only partially suppressed STAT3 phosphorylation, whereas the combination of both treatments completely attenuated STAT3 phosphorylation.

### 3.6. Ginkgolide B Abolished JAK2/STAT3 Phosphorylation Induced by LPS

**Figure 6:**
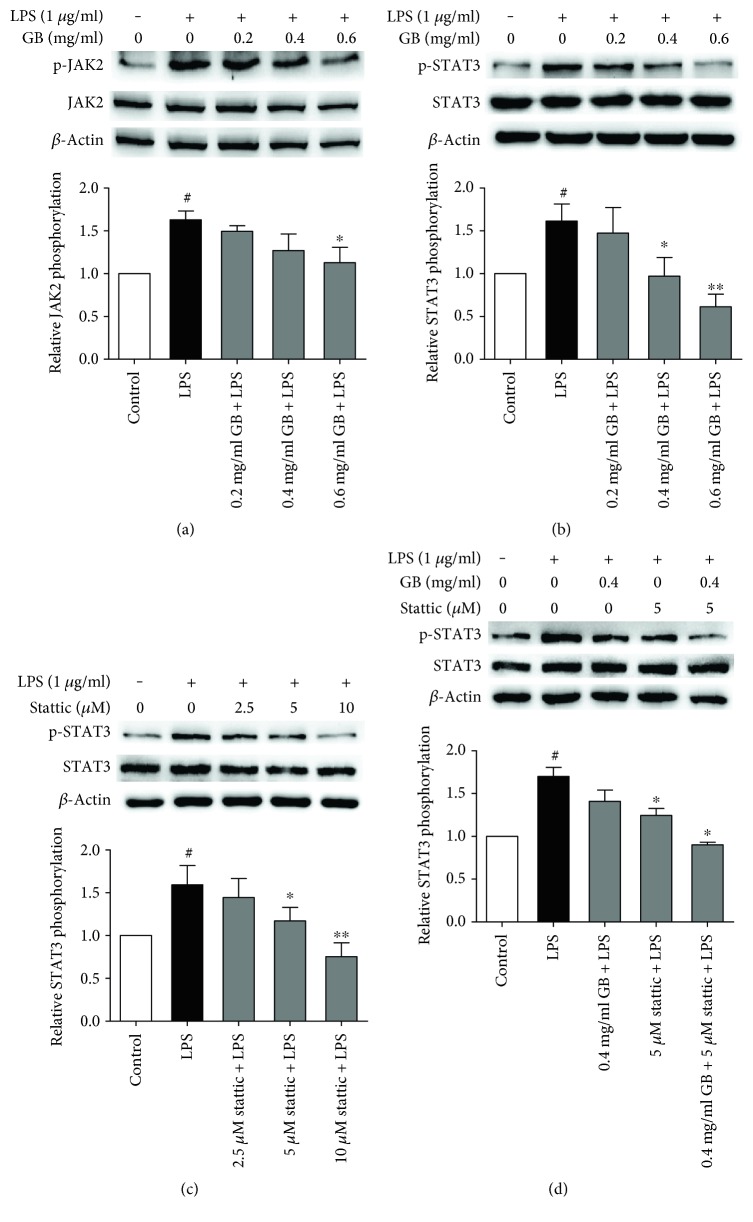
Ginkgolide B inhibits JAK2 and STAT3 phosphorylation in LPS-treated HUVECs. The cells were treated with various concentrations of ginkgolide B for 1 h, and LPS was added for another 8 h. The column chart represents the density analysis from four independent experiments. (a) Ginkgolide B inhibited JAK2 phosphorylation that was induced by LPS. (b) Ginkgolide B inhibited STAT3 phosphorylation that was induced by LPS. (c) Stattic inhibited STAT3 phosphorylation that was induced by LPS. (d) The combination of ginkgolide B and stattic synergistically inhibited STAT3 phosphorylation that was induced by LPS. ^#^*p* < 0.05, significant difference between non-LPS-treated cells and LPS-treated cells; ^∗^*p* < 0.05, ^∗∗^*p* < 0.01, significant difference between non-ginkgolide B-treated cells and ginkgolide B-treated cells.

To further confirm the suppressive effect of ginkgolide B on JAK2/STAT3 phosphorylation is associated with TLR4 signal, we used the TLR4-specific agonist LPS. As shown in Figures 6(a) and 6(b), LPS increased JAK2 phosphorylation by 62.9% and STAT3 phosphorylation by 61.4%, and ginkgolide B dose dependently abolished JAK2 and STAT3 phosphorylation. Furthermore, we determine the effect of STAT3 inhibitor stattic on STAT3 phosphorylation in LPS-treated HUVECs. As shown in Figure 6(c), stattic (2.5, 5, and 10 *μ*M) dose dependently inhibited STAT3 phosphorylation that was induced by LPS. Additionally, we also evaluated whether combined stattic and ginkgolide B synergistically inhibits STAT3 phosphorylation. As shown in Figure 6(d), the low concentration of stattic (5 *μ*M) or ginkgolide B (0.4 mg/ml) alone could not completely suppress STAT3 phosphorylation; however, the combination of both completely attenuated STAT3 phosphorylation induced by LPS.

### 3.7. Ginkgolide B Inhibited p38 MAPK Phosphorylation in High Glucose-Stimulated HUVECs

**Figure 7:**
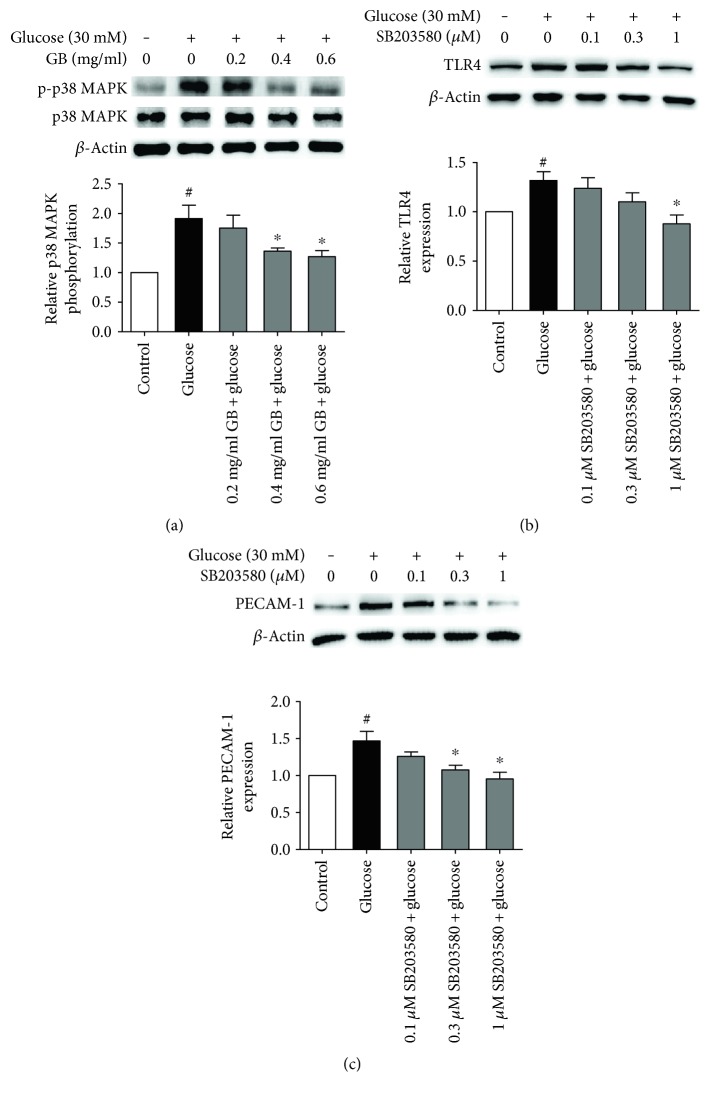
Ginkgolide B inhibits p38 MAPK phosphorylation in high glucose-treated HUVECs. The cells were treated with various concentrations of ginkgolide B for 1 h, and glucose was added for another 8 h. The phosphorylation of p38 MAPK was analyzed by Western blot. The column chart represents the density analysis from four independent experiments. (a) Ginkgolide B inhibited p38 MAPK phosphorylation that was induced by high glucose. (b) SB203580 inhibited TLR4 expression that was induced by high glucose. (c) SB203580 inhibited PECAM-1 expression that was induced by high glucose. ^#^*p* < 0.05, significant difference between nonhigh glucose-treated cells and high glucose-treated cells; ^∗^*p* < 0.05, significant difference between non-ginkgolide B-treated cells and ginkgolide B-treated cells.

The MAPK family has garnered significant attention because of its vast implications in signaling and crosstalk with other signaling networks. To explore the underlying mechanisms of action of ginkgolide B on TLR4 activation, we investigated the effect of ginkgolide B on p38 MAPK phosphorylation that was induced by high glucose. As shown in Figure 7(a), p38 MAPK phosphorylation was enhanced by 91%, and ginkgolide B (0.4 and 0.6 mg/ml) potently decreased its phosphorylation in high glucose-treated cells. To further investigate whether the p38 MAPK has regulatory action on TLR4 activation, we use p38 MAPK inhibitor SB203580. Our preliminary experiment showed that SB203580 (0.1, 0.3, and 1 *μ*M) could inhibit p38 MAPK phosphorylation in high glucose-treated HUVECs (data not shown). As shown in Figures 7(b), and 7(c), SB203580 (0.1, 0.3, and 1 *μ*M) dose dependently decreased TLR4 and PECAM-1 expression in glucose-treated HUVECs and 1 *μ*M SB203580 completely inhibited their expression.

## 4. Discussion

Immune dysfunction plays a key role in chronic inflammation, including diabetes and atherosclerosis [29, 30]. Early studies demonstrated that TLR activation was induced by bacterial infection that was caused by bacterially secreted LPS. Toll-like receptors are pattern-recognition receptors that play a role in the host defense against invading microbial pathogens. Accumulating evidence indicates that TLRs are involved in diabetic complications [31, 32]. Ahmad et al. recently reported that subjects with type 2 diabetes had significantly elevated mRNA levels of TLR2 and TLR4 compared with nondiabetic obese subjects. They also found a strong association between TLR expression and cytokines (IL-6 and tumor necrosis factor *α*) in peripheral blood mononuclear cells [33]. In the present study, we found that ginkgolide B inhibited the TLR4-mediated inflammatory response in high glucose-treated HUVECs. High glucose treatment increased the expression of TLR4 and MyD88 and increased the levels of the inflammatory proteins PECAM-1, IL-6, and MCP-1. Ginkgolide B suppressed the expression of these inflammatory proteins that was induced by high glucose. Moreover, ginkgolide B inhibited LPS-induced TLR4 and PECAM-1 expression, suggesting that ginkgolide B suppresses the TLR4-mediated inflammatory response that is induced by high glucose in HUVECs.

Ginkgolide B is a PAF receptor antagonist. We evaluated whether the action of ginkgolide B is associated with PAFR blockade in high glucose-stimulated HUVECs. Immunoprecipitation revealed that high glucose stimulation increased TLR4 and PAFR expression and enhanced the binding between PAFR and TLR4. Ginkgolide B attenuated these effects. This suggests an interaction between PAFR and TLR4 in high glucose-stimulated HUVECs. We also performed a functional analysis using siRNA. PARF knockdown with PAFR siRNA reduced TLR4 expression, and knockdown with TLR4 siRNA decreased PAFR expression in nonhigh glucose-treated cells and high glucose-treated cells. Our findings demonstrated an interaction between TLR4 and PAFR activation in high glucose-treated HUVECs. The inhibition of high glucose-induced TLR4 activation by ginkgolide B appears to be associated with PAFR suppression. These results are consistent with a previous study that showed that the absence of TLR4 greatly diminished pulmonary inflammation and the same phenotype in PAFR^−/−^ animals. Both TLR4 and PAFR may influence lung inflammation that is induced by lipoteichoic acid (LTA; a major outer cell wall component of Gram-positive bacteria) either by sensing LTA directly or through the recognition and signaling of endogenous mediators that are induced by LTA [34].

Growing evidence indicates that the JAK2/STAT3 pathway plays an important role in tumors and inflammation. Some studies have shown that STAT3 is involved in the IL-6-mediated inflammatory response [35]. We found that JAK2/STAT3 phosphorylation was enhanced in high glucose- and LPS-treated HUVECs, and ginkgolide B attenuated their phosphorylation. This suggests that JAK2/STAT3 activation is involved in the high glucose-induced inflammatory response, and the inhibition of TLR4 signaling by ginkgolide B might be associated with the suppression of JAK2/STAT3 phosphorylation. p38 MAPK plays a critical role in cell activation, including inflammation [36–38]. The present results showed that ginkgolide B might suppress p38 MAPK phosphorylation that is induced by high glucose. The p38 MAPK inhibitor SB203580 also decreased the expression of TLR4 and PECAM-1. This suggested that p38 MAPK might be involved in positive feedback loop with TLR4 signaling and ginkgolide B inhibited the process.

## 5. Conclusion

Ginkgolide B suppressed the expression of the TLR4-mediated inflammatory response and inhibited the binding of PAFR and TLR4 in high glucose-treated HUVECs. The mechanism of action of ginkgolide B appears to be associated with blockade of the JAK2/STAT3 pathway and p38MAPK phosphorylation. Our study provided evidence that PAFR inhibition might be considered as a therapeutic strategy for diabetic vascular inflammation.
